# Mating Behavior of *Rosalia batesi* (Coleoptera: Cerambycidae) Is Mediated by Male-Produced Sex Pheromones

**DOI:** 10.3390/insects9020048

**Published:** 2018-04-23

**Authors:** Satoshi Kiriyama, Ryûtarô Iwata, Midori Fukaya, Youtaro Hoshino, Yasuyuki Yamanaka

**Affiliations:** Laboratory of Forest Zoology, Department of Forest Science and Resources, College of Bioresource Sciences, Nihon University, Fujisawa, Kanagawa 252-0880, Japan; kiriyama.satoshi@nihon-u.ac.jp (S.K.); viridisetviridis@gmail.com (M.F.); charichari830@outlook.jp (Y.H.); y_yamanaka365@yahoo.co.jp (Y.Y.)

**Keywords:** Cerambycidae, Rosaliini, longhorned beetle, male sex pheromone, calling behavior, push-up stance

## Abstract

*Rosalia batesi* Harold (Cerambycidae) is a hardwood boring species endemic to Japan. We investigated the adult mating behavior of this species in the field and laboratory. Most males appeared on mating sites before noon, significantly earlier than females did, in field observations. The female approached and contacted the male; the male responded and started the successive mating sequence, comprising mounting, copulation, and appeasement behavior. Before the encounter, the male raised its fore and mid legs and bent the abdominal tip ventrally. Next, a peculiarly structured bifurcate tip was exposed with opening and closing motion, which can be observed in the entire family Cerambycidae and is thought to be associated with the emission of volatile male sex pheromones. Male and female orientation toward conspecifics was examined using T-shaped olfactometers in four combinations (male–male, female–male, female–female, male–female). Males exclusively attracted females, indicating the existence of male-produced sex pheromones. A laboratory bioassay with three temperature regimes revealed the temperature dependence of this calling behavior. The calling behavior occurred only when the air temperature and male body surface temperature, which are associated with light intensity, were within the range of 26–33 °C and 26–28 °C, respectively.

## 1. Introduction

The chemical ecology and reproductive behavior of Cerambycidae have been studied mainly in pest species after the 1980s [1,2,3,4,5,6]. However, the reproductive behavior of non-pest cerambycid species that live in dead wood during their larval phase has not been well studied, except for a few *Xylotrechus* species [7].

The genus *Rosalia* (Cerambycidae: Rosaliini) is distributed world-wide (Holarctic and Oriental), and most species are large and have a spectacular appearance. The well-studied European species, *R. alpina* (Linnaeus), is a protected species in several countries [8,9]. The Japanese endemic longicorn beetle, *R. batesi,* is distributed over most of Mainland Japan and has an important function of decomposing dead hardwood in the Japanese forest ecosystem. This species is very popular in Japanese culture, owing to its spectacular appearance and color. It often appears on postage stamps and on the covers of books and magazines. Iwata et al. [10] reported the ecological function of *R. batesi*; however, the mating behavior and the communication between males and females have not yet been investigated in detail. The mating behaviors of cerambycid species vary with the lifecycle and host characteristics [11,12]. This species is known to exhibit a very peculiar biology: larvae bore deep into heartwood of hardwood [10] and possess only a much reduced larval bacterial gut flora when compared with other cerambycid species (H. Ueda et al., unpublished). Moreover, despite global warming, *R. batesi* was reported to have expanded its distribution area toward the lowlands in central Japan [13]. Taken together, this species shows peculiar physiological and ecological characteristics. Investigating the reproductive behavior of *R. batesi* may provide new insights into the chemical ecology of the family Cerambycidae.

Hanks [11] initially suggested that the presence or absence of long-range pheromones in Cerambycidae depends on the chance of encountering a member of the opposite sex. Species that live in living trees need long-range pheromones, because their host plants are amply distributed, and thus the chance of encounter may be small. In contrast, species that live in dead wood do not need long-range pheromones, because dead wood is a limited resource in the forest, and therefore the chance of encounter is high. However, this hypothesis was amended. To date, whether or not Cerambycidae use pheromones is thought to depend on the phylogeny, rather than on the host resource availability [14]. In the subfamily Prioninae, females emit volatile sex pheromones [15], whereas in the subfamilies Cerambycinae, Spondylinae, and in *Monochamus* spp. and *Anoplophora glabripennis* of the subfamily Lamiinae, males express aliphatic volatile sex (or aggregation) pheromones [16]. Therefore, we would expect *R. batesi* males to use male pheromones.

Recent studies have shown that the composition and structure of pheromones in congeneric species of Cerambycidae are similar, especially in *Monochamus* and *Xylotrechus* [6,17,18]. Male and female adults of *Rosalia funebris* have been observed to be attracted to a synthetic chemical ingredient in paint [19]. Ray et al. [20] suggested that the reason for this attraction is that the paint component is an analog of *R. funebris*’ aggregation pheromone. The *R. funebris* aggregation pheromone has now been identified as (*Z*)-3-decenyl (*E*)-2-hexenoate [21]. However, the anatomical source of the aggregation pheromone has not yet been identified. In Cerambycidae adults, male pheromones are often emitted from gland pores on the pronotum, which also emit sex (or aggregation) pheromones [20]. However, this anatomical structure has not been found in *R. funebris* [20]. In the European congeneric species *R. alpina*, males exhibit a peculiar behavioral pattern, which is suggestive of a territoriality system [22], suggesting the presence of male pheromones. The main component of the volatile male “aggregation–sex pheromones” (term after Cardé [23]) of *R. alpina* has been identified as 3,5-dimethyl-6-(1-methybutyl)-pyran-2-one, an alkylated pyrone [24]. In this species, the host (*Fagus sylvatica*) volatile component, alone or in combination with the pheromone, exhibited little attractive activity [24].

Since the end of the last century, *R. batesi* has been expanding in central Japan [13,25]. This expansion supplies us with a large number of individuals to study in field observations and laboratory experiments. In our preliminary observation of *R. batesi*, we noticed that females approached males without direct contact. This may indicate that the male releases volatile sex pheromones to attract the female.

Males of the *R. batesi* species show a rather broad variation of body length [10]. In some cerambycid species, male adult beetles exhibit different mating strategies with different body length: smaller males are more sensitive than larger males to cues emitted by the female [5,26]. Therefore, we considered male body length as a parameter in our mating experiments.

In this work, we aimed at confirming which sex releases and which sex responds to pheromones, identifying the time of day at which adults appeared, describing the calling posture putatively associated with sex pheromone emission, and clarifying the mating sequence of *R. batesi*. Moreover, we tried to identify the anatomical location of the pheromone releasing organ. To verify the existence and determine the effect of the male sex pheromones, we conducted a bioassay using a T-shaped tube with live females or males as lures. We also showed the importance of ambient and body surface temperatures, which influence and trigger the conspicuous calling posture of males.

## 2. Materials and Methods

### 2.1. Animals

Adult *R. batesi* were collected from rotten logs and dead wood in (1) Onojimachi, Machida, Tokyo Prefecture (from 27 June to 14 July 2009, from 25 June to 15 July 2010, and on 29 July 2012); (2) Ryôkamisusuki and Nagaru, Ogano, Saitama Prefecture (on 7 July 2010); (3) Nagatsuta, Midori-ku, Yokohama, Kanagawa Prefecture (on 23 June 2010 and 7 July 2010); (4) Kanosawa, Ôana, and Fujiwara-Takaragawa, Minakami, Gunma Prefecture (from 6 August to 10 August 2010, and on 21 August 2012); and (5) Hanasaki and Tokura, Katashina, Gunma Prefecture (on 9 August 2010 and 21 August 2012), all of which are in Central Japan. In Machida, the beetles aggregated on piled large rotten logs of various hardwood species. *Ailanthus altissima* (Mill.) Swingle was the main tree host. Starting from 2008, we also kept *A. altissima* logs in an outdoor cage near the laboratory and harvested newly emerged adult beetles on 24 June 2009, and on 30 June, 1 July, 2 July, and 14 July 2010. In the laboratory, the beetles were kept in individual plastic cases (ca. 90 × 90 × 90 mm). The plastic cases contained a cotton ball soaked in sucrose solution to feed the beetles before being subjected to the bioassay. The sucrose-soaked cotton balls were exchanged every 3 days. For the pheromone assay, animals served either as an odor-source animal or as an odor-reacting (test) animal.

### 2.2. Field Observations on the Appearance of Adult Beetles

In Machida (Location (1); 3 July, 16 July, and 19 July 2008), the time that each of the individual beetles was collected was recorded (mostly between 09:00 and 14:00). The results were sorted in order to detect the sexual difference of the appearance time zone of the day.

### 2.3. Laboratory Observations of Sexual Attraction and Subsequent Mating Behavior

The body lengths of all adult beetles subjected to the laboratory bioassay and the dried specimen were measured using electric calipers (Mitutoyo, Kawasaki) (males: 24.51 ± 4.31 mm, *n* = 195; females: 24.68 ± 3.18 mm, *n* = 110). The adults supplied to the laboratory bioassay were then divided into four groups, separated by sex and body size (large males: larger than the average male length; small males: smaller than the average male length; large females: larger than the average female length; and small females: smaller than the average female length). All adult beetles that were subjected to the bioassay had mated at least once during the preliminary observations. Adult male and female beetles of this species are known to mate multiple times with different partners. Therefore, subjecting an adult to multiple trials of the mating bioassay is not expected to bias the results. Thus, some specimens were subjected to the bioassay more than twice.

We eliminated individuals that did not show any positive response to any individual of the opposite sex from the bioassay while preliminarily checking each of the beetles. For each observation, the selected male and female specimens were placed simultaneously in a plastic container (290 × 190 × 170 mm) and their behavior was observed for 60 min. During this time, the following parameters were recorded: (a) the time until the first mounting took place, (b) the total duration of copulations, (c) the total duration of mountings, and (d) the duration of individual copulations. In addition, details of the behavior of the pair and the sequence of male calling behavior were recorded. Our terminology follows that of Michelsen [27,28,29] for the repertoires of sexual behavior of cerambycid adults—namely, licking, tapping, and biting. In the case of attacks against female antennae, these three behavioral items were not discriminated, because the attacks were vigorous and swift.

### 2.4. Laboratory Bioassay Using a T-Shaped Olfactometer

**Figure 1:**
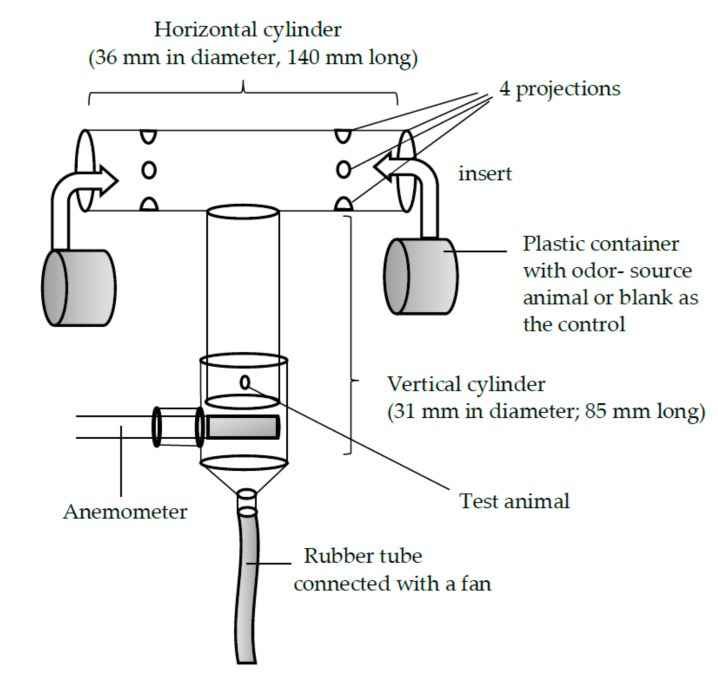
The T-shaped glass olfactometer used in the experiment with *Rosalia batesi*. Of the two arms, the one that the test animal selected was recorded.

A T-shaped glass olfactometer (Figure 1), which was laid on the table, was used to investigate the beetles’ olfactory orientation behavior when choosing between two odor sources. Each arm of the olfactometer had four protrusions that extended from the inner wall, 45 mm from the ends of the cross cylinder. These protrusions served as anchors for the partially perforated plastic container in which the odor-source animal was placed. The diameter of the plastic container was slightly smaller than that of the horizontal glass cylinder. Thus, the container could be easily inserted into and fixed inside the horizontal cylinder. To the vertical cylinder of the olfactometer, another glass tube with a slightly greater diameter than that of the vertical cylinder of the olfactometer was attached. The air in the cylinder was sucked out by a pump connected to the end of the rubber tube. The airflow was adjusted to be 0.45 m/s, using an anemometer. The container of the odor-source animal was opaque, so the test animal would not be influenced by visual cues. While the odor-source animal was placed at the right or left arm, the control (the empty plastic container) was placed at the opposite arm. For each of the four possible combinations of odor source (S) and test animal (T) sexes—(1) S-male vs. T-male, (2) S-female vs. T-male, (3) S-female vs. T-female, and (4) S-male vs. T-female—50 trials were performed. After each trial, the whole olfactometer was submerged into a solution of 75% ethanol, washed, and dried thoroughly. The position (right or left) of the test animal was alternated between trials in order to offset the influences of direction and lighting condition. The test animal was considered inactive if it did not arrive at either end of the arm within 10 min of the beginning of the trial. All experiments were carried out at room temperature (26.2 °C ± 0.5 **°**C; mean ± S.D.) and at a luminous intensity of 2000 Lux.

### 2.5. Laboratory Observation of Male Calling Behavior with Different Light Intensity and Temperature Regimes

**Figure 2:**
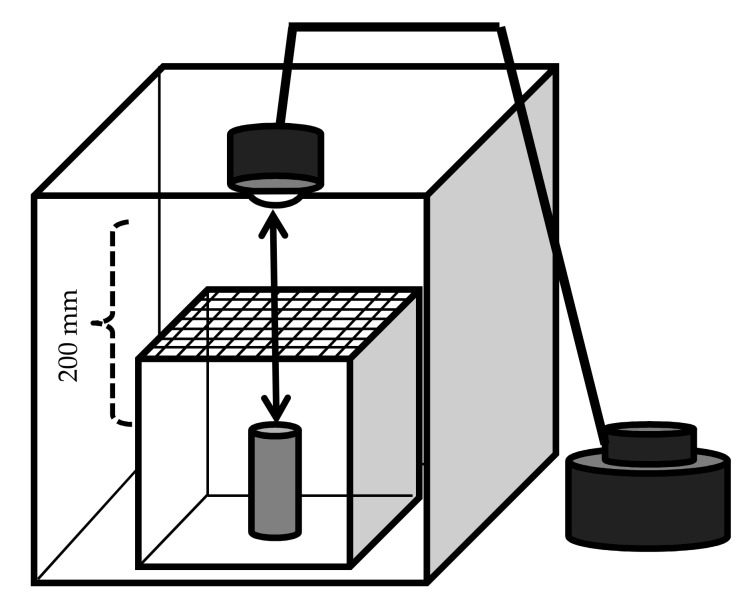
Experimental device for the detection of the influence of temperature and luminous intensity on the calling behavior of *Rosalia batesi* males. A corrugated cardboard box (400 × 400 × 400 mm) was prepared, in which a glass chamber (150 × 150 × 150 mm, with a nylon mesh on top) containing a perpendicular *Zelkova serrata* bolt (80 mm long, 40 mm in diameter) was installed. An incandescent lamp was attached to the ceiling of the cardboard box (200 mm from the bolt top). The incandescent lamp was powered with three different voltages (100, 50, and 30 V) to achieve three temperature and luminous intensity regimes (high, medium, and low, respectively). The test animal (*Rosalia batesi* male) was released near the bolt.

Male calling behavior was observed in the laboratory as depicted in Figure 2. A cardboard box (ca. 400 × 400 × 400 mm) was prepared as follows: one of the four lateral walls was removed so that the behavior of the animals inside the box could be observed. A glow lamp (SB416B; 110 V, 40 W; Panasonic, Osaka) was attached to the top of the box. The glow lamp was connected to a slide transformer with alternating current (RSA-10; Tokyo Rikosha Co., Ltd., Saitama, Japan) to adjust the luminous intensity. A glass box with a detachable nylon mesh (1.5 mm) at the top was placed into the cardboard box beneath the lamp (150 × 150 × 150 mm). In the center of the glass chamber, a *Zelkova serrata* (Thunb.) Makino bolt (ca. 80 mm high, 40 mm in diameter; with intact bark) was installed perpendicularly. The incandescent lamp was powered with three different voltages (100, 50, and 30 V) to achieve three different light and temperature regimes (a, b, and c): the luminous intensities and temperatures at the top and bottom of the bolt were measured using the Illuminance UV Recorder, TR-74Ui (T and D Corporation, Nagano, Japan), and were (a) 5040 and 2500 Lux, respectively; (b) 310 and 185 Lux, respectively; and (c) 63 and 45 Lux, respectively. The air temperatures of these three regimes decrease in this order (see Results, Section 3.6). Under each regime, 20 trials of 30 min were carried out. The bioassay was conducted as follows: An *R. batesi* male was released on the bolt. The animal’s behavior was recorded with a video camera (HDC-TM45; Panasonic, Osaka, Japan). The times of the beginning and the end of the calling behavior, the air temperature inside the chamber, and the location of the male were recorded. The locations of the males were classified into one of five categories: (1) the top of the bolt, (2) the upper half of the bolt (above the midpoint), (3) the bottom half of the bolt (below the midpoint), (4) the floor of the chamber, or (5) the ceiling of the chamber and other locations. The first four (1–4) locations were assumed to have descending luminous intensity, based on the measurements conducted at the top and bottom of the bolt. After 30 min, the body surface temperature of the male beetle was measured with a non-contact Handy Thermometer (IT2-80; Keyence, Osaka, Japan) and an infrared thermograph (i7; FLIR, Wilsonville, Oregon, USA).

The durations of the calling behavior were analyzed in relation to the temperature and luminosity. Calling behavior durations in the three regimes were compared using the Kruskal–Wallis test in combination with the Steel–Dwass multiple test. Correlations between calling behavior duration, air temperature of the cage, and beetle body surface were analyzed using the Spearman’s rank correlation test.

### 2.6. Statistical Analyses

All statistical analyses were performed with a free version of “R”, except for χ^2^ tests, which were performed using “Excel”.

## 3. Results

### 3.1. Field Observations of the Appearance of Adult Beetles

**Figure 3:**
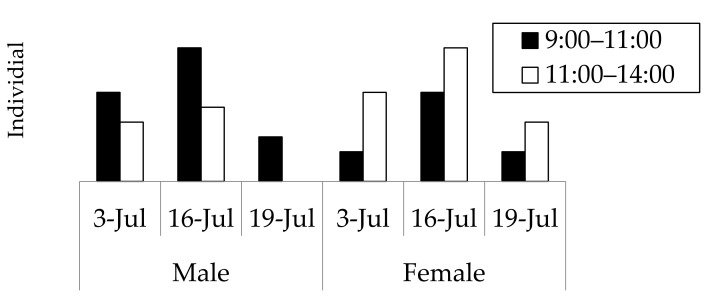
Numbers of *Rosalia batesi* adult individuals observed in the suburban forested area in Onojimachi, Machida, Tokyo Pref., Japan. (3 July, 16 July, and 19 July 2008). Males appeared significantly earlier than females (χ^2^ test, *p* < 0.05).

**Figure 4:**
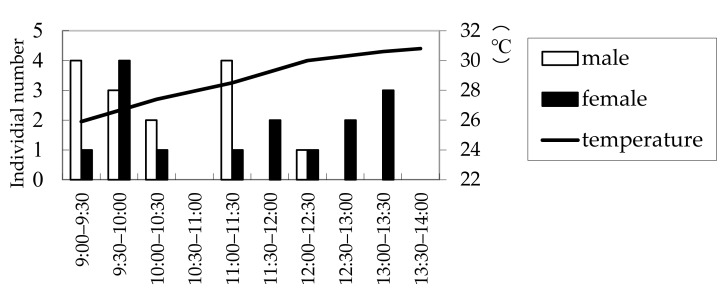
Numbers of *Rosalia batesi* adult individuals observed in the suburban forested area in Onojimachi, Machida, Tokyo Pref., Japan. (16 July 2008). Males appeared significantly earlier than females (Mann–Whitney *U* test, *p* < 0.05).

The results of the field observations performed on 3 July, 16 July, and 19 July 2008, at a wood deposit in Onojimachi, Machida, Tokyo Pref., are shown in Figure 3. For all three days, males abounded in the morning (09:00–11:00), whereas females continuously appeared from 09:00 to 14:00. However, more females appeared after 11:00 than before 11:00, indicating that males appeared significantly earlier than females (χ^2^ = 4.5773 > χ^2^_0.05_ = 3.84, d.f. = 1, *p* < 0.05). The day on which the greatest number of beetles (29 beetles) was observed was 16 July, for which males’ and females’ appearance times were further compared with a more accurate test. The result was consistent with that of the three-day combined χ^2^ test (Mann–Whitney *U* test, |*z*| = 2.10, *p* < 0.05) (Figure 4).

### 3.2. Durations of Mounting and Copulation in Relation to Body Lengths of Males and Females

**Figure 5:**
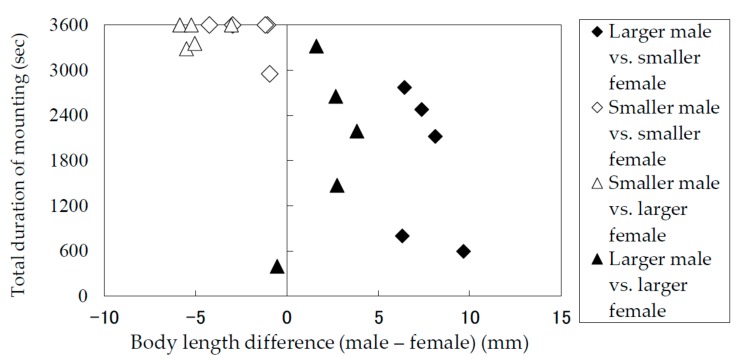
Total mounting durations of *Rosalia batesi* adult pairs with different body lengths (male body length—female body length).

**Figure 6:**
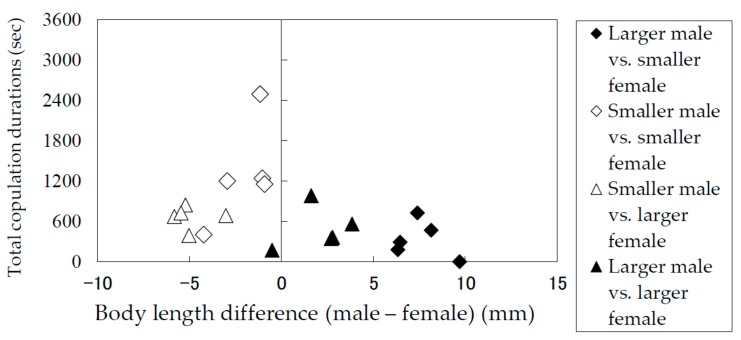
Total copulation durations of *Rosalia batesi* adult pairs with different body lengths (male body length—female body length).

The results of the laboratory observation of the mating behaviors are summarized in Table 1. The total mounting durations (copulation duration included) (Figure 5) and the total copulation durations (Figure 6) of the pairs were recorded and analyzed in terms of the difference between the body lengths of the pair. Males and females were divided into groups of smaller than average and larger than average individuals. Figure 5 and Figure 6 indicate that smaller males performed longer mounting and copulation, irrespective of the body length of their mates. This tendency was significant (Mann–Whitney *U* test) in terms of both mounting duration (|*z*| = 3.71, *p* < 0.05) and copulation duration (|*z*| = 3.66, *p* < 0.05). In addition, only smaller males were observed to embrace females with their hind legs crossed on the female’s sternum to fix and subdue her.

### 3.3. Ethogram of Mating Behavior

**Figure 7:**
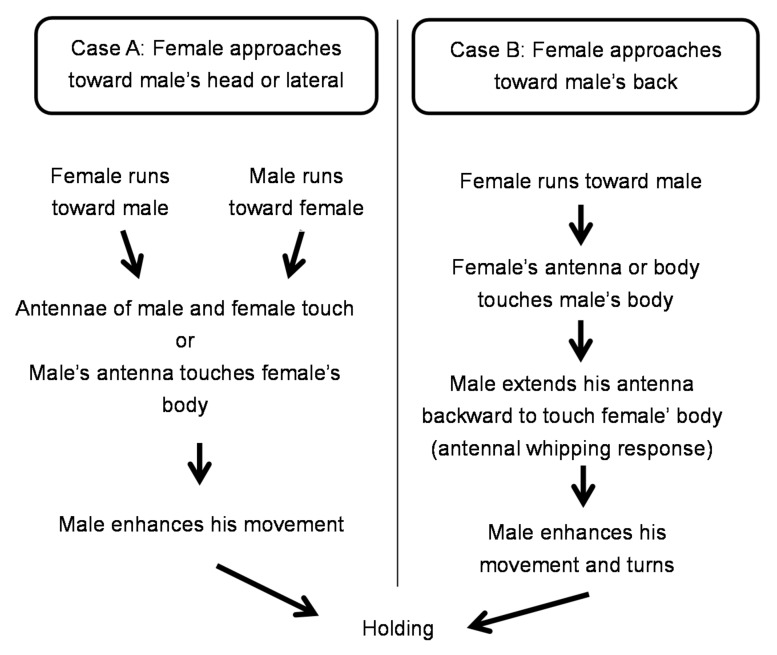
Ethogram of *Rosalia batesi* male and female toward mating.

Encounters generally began with the female approaching the male. Case A summarizes cases in which the female approached the male from his lateral or frontal side. Case B summarizes cases in which the female approached the male from behind (Figure 7). In Case A, the female and the male approached each other simultaneously. Then, either the antennae of both partners touched or the male’s antenna touched the female’s body. This prompted the male to hold the female and mount her. In Case B, the female approached the male, and her antenna, leg, or mouth touched the male’s body or legs. This led to the male’s “antennal whipping response” in which the antennae whipped from the front to the back. This resulted in the male’s antenna making contact with the female’s body, the male recognizing the presence of the female, and then mounting her.

After the holding (firm contact), a behavioral sequence under the male’s leadership took place. Various behavioral actions of “appeasement” by males toward females were observed in all cases. The details and frequencies of these appeasement behaviors are presented in Table 2.

In the non-copulation condition (mounting but not copulating), the male exhibited a slow licking behavior of the female’s labrum, head vertex, pronotum, scutellum, and elytral bases. In the copulation condition (mounting and copulating), the male exhibited a back-and-forth movement of the abdominal tip in addition to the licking behavior, which was synchronized with pulling of the female’s genitalia. Additionally, this rhythmical abdominal movement was synchronized with tapping of the male’s mouth against the female’s head vertex, pronotum, and elytra. Furthermore, males often attacked the basal portion of the female’s antenna using their mandibles, pressing it down or fixing it to prevent spontaneous movement from the female (antenna-biting). The antenna-biting behavior took place significantly more frequently in the non-copulation condition than in the copulation condition (χ^2^ = 101.81 > χ^2^_0.05_ = 3.84, d.f. = 1, *p* < 0.05), especially directly after the cessation of copulation or upon the female’s denial of mounting (running away, pressing the sternum on the bolt by stretching the legs, bending down the abdomen, or kicking the male). We considered this antenna-biting behavior to promote mounting and copulation, by tranquilizing the female’s arbitrary movement. These observations led us to call this series of male behavior appeasement. The so-called antennation behavior [30,31], a sword-battle-like contact of the antennae of male and female, was not observed in this species. Scratching behavior against the female pronotum was observed in one very large male of more than 33 mm body length (Table 1, M3; Table 2).

**Figure 8:**
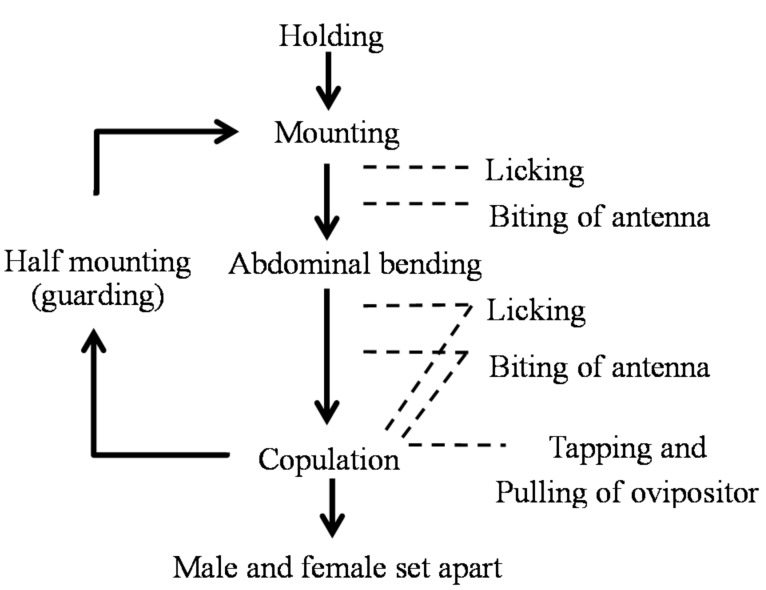
Ethogram of *Rosalia batesi* male and female in mating after firm contact.

Based on the above observations, we present ethograms to summarize the behavioral sequences of adult *R. batesi* from the encounter of the male and female to holding (Figure 7), and from holding to the cessation of contact after copulation (Figure 8).

### 3.4. Male Behavior in the Push-Up Stance (Calling Behavior) and the Structure of the Male Abdominal Tip

**Figure 9:**
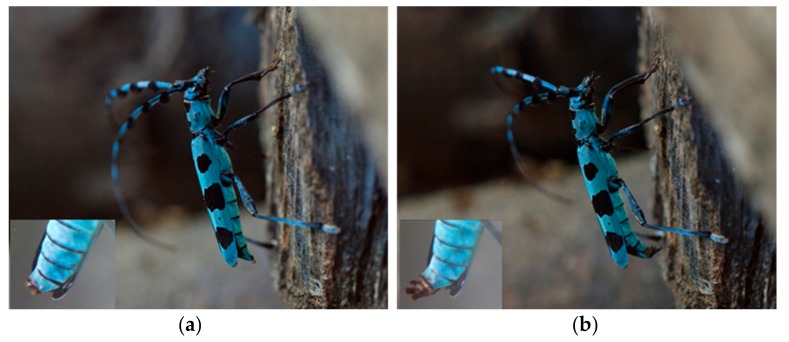
Two phases of *Rosalia batesi* male’s push-up stance. (**a**) Motionless phase, in which only the tip of the eighth tergite is visible; (**b**) Putative pheromone-emission phase, in which the male exposed the peculiar bifurcate abdominal tips, which he opened and closed in a rhythmic motion with 3–4 s intervals. The male switched between these two phases semi-periodically (photo by Takehiko Sato).

**Figure 10:**
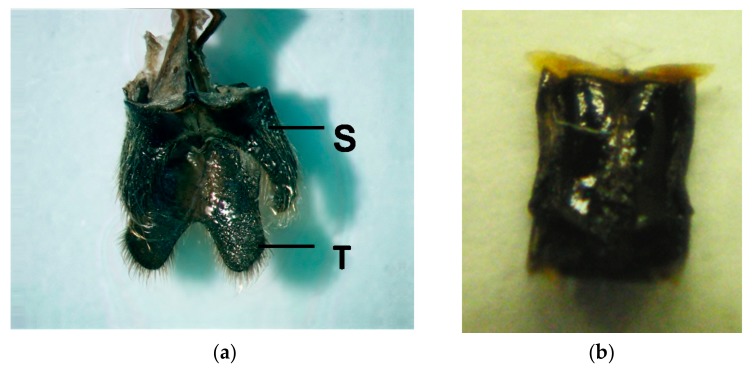
(**a**) The eighth abdominal tergite (T) and sternite (S) of an adult male *Rosalia batesi*. The tergite and sternite are membranous rather than chitinized, and are of a peculiar bifurcate shape, with numerous sensory apertures on the entire surface of the tergite. (**b**) The eighth abdominal of the adult female *Rosalia batesi* has a much simpler appearance.

While observing adult *R. batesi* mating behavior in the laboratory, we frequently observed a push-up stance performed by male beetles. In this stance, the male occasionally extended his fore and mid legs to raise up his body. This stance consisted of two phases (Figure 9): the motionless phase and the putative pheromone-emission phase. In the putative pheromone-emission phase, the male exposed his peculiar bifurcate abdominal tips (Figure 10), which he opened and closed in a rhythmic motion with 3–4 s intervals. The male switched between these two phases semi-periodically. This posture was thought to be associated with the emission of volatile pheromones to attract conspecifics, and thus was termed calling behavior.

**Figure 11:**
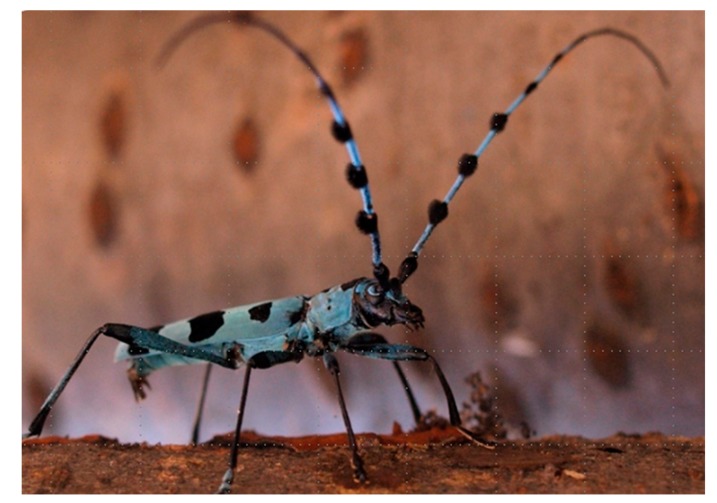
“Push-up stance” of *Rosalia batesi* male adult, with the abdominal tip raised up.

This behavior partly resembles that observed in *Neoclytus acuminatus* [32]. However, different from *Neoclytus acuminatus,* the *R. batesi* male’s push-up stance brings its body to an almost parallel position to the substratum, and his abdominal tip is strongly bent downward (Figure 11). Usually, the eighth abdominal tergite and sternite (Figure 10a; [33,34]) are retracted within the body cavity, except for the very tip of the tergite. During the push-up stance, the two bifurcate structures are wholly exposed from the bent abdominal tip (Figure 11). This behavior and anatomical structure are thought to be associated with pheromone emission to attract females, similar to the prionine *Prionus californicus* female [15,35]. In contrast, the female’s abdominal tip is simple and membranous (Figure 10b), and is therefore thought to be unassociated with pheromone emission.

### 3.5. Male and Female Choices in the T-Shaped Olfactometer

The results of the T-shaped olfactometer bioassay are presented in Table 3. Among the four combinations of odor source (S) and test animals (T), namely, (1) S-male vs. T-male, (2) S-female vs. T-male, (3) S-female vs. T-female, and (4) S-male vs. T-female, only the last combination (4) showed a significant difference. The female was attracted to male in 42 out of 50 trials, and this bias was significant (χ^2^ = 31.39 > χ^2^_0.001_ = 10.83, d.f. = 1, *p* < 0.001). In the other three combinations, no significant bias of moving toward the odorsource was observed (*p* > 0.05). Thus, we concluded that females were attracted to the volatile odor emitted by males only. We assumed that this putative odor is a sex pheromone rather than an aggregation pheromone. Moreover, most of the test females in experiment (4) became motionless when they reached the odor source (male) in the plastic container. In contrast, most test females in experiments (3), and the misguided test females in experiment (4) having arrived at the plastic container without a male (i.e., the control or another female as the odor source), continued moving restlessly.

### 3.6. Effect of Temperature and Light Intensity on Male Calling Behavior

**Figure 12:**
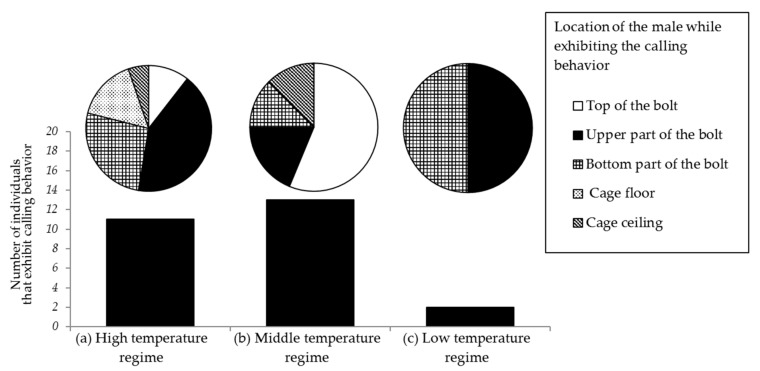
Numbers of trials in which the male *Rosalia batesi* exhibited calling behaviors. Experiments were conducted in a laboratory cage (150 × 150 × 150 mm) equipped with a *Zelkova serrata* bolt (80 mm in length, 40 mm in diameter) set perpendicularly on the floor, under three light and temperature regimes (high, middle, and low). The male beetles’ positions while exhibiting calling behaviors and their frequencies are shown in the pie charts.

The laboratory bioassay of male calling behavior with three different light and temperature regimes ((a), (b), and (c)) was carried out with 20 trials for each regime (Figure 12). Body and room temperature in the different regimes were dependent on the illumination intensity, and the temperature increased as the time elapsed. Regime (a) started at 26.4 °C ± 0.6 °C and ended at 33.2 °C ± 0.5 °C, regime (b) started at 26.0 °C ± 0.3 °C and ended at 27.4 °C ± 0.3 °C, and regime (c) started at 26.0 °C ± 0.5 °C and ended at 26.0 °C ± 0.3 °C. In the high temperature regime (a), most beetles exhibited calling behaviors with the push-up stance on the lateral side of the *Zelkova* bolt (upper or bottom part) and on the floor (Figure 12). Males seemed to avoid very bright spots, such as the top of the bolt, where they stayed only very shortly. When the air temperature in the cage rose to 33 °C, most beetles took refuge in a shady spot and remained motionless. In the mid temperature regime (b), most beetles were observed to stay in bright spots.

The body surface temperatures of the male beetles that exhibited calling behaviors were 30.3 °C ± 0.9 °C in regime (a) (*n* = 11), 27.6 °C ± 0.6 °C in regime (b) (*n* = 13), and 25.7 °C in regime (c) (*n* = 2). The average durations of the calling behavior in regimes (a) and (b) were long (70 and 133.25 s, respectively). In the low temperature regime (c), a general recess in activity was noticed and very little calling behavior was observed (13.9 s). The durations of the calling behavior in the three regimes were significantly biased (Kruskal–Wallis test, *H*′ = 12.236, *p* = 0.0022), and the values were significantly different from each other (*p* < 0.05), except between (a) and (b) (*p* > 0.05) (Steel–Dwass test).

The correlation between the duration of the calling behavior and the air temperature in the high temperature regime (a) was significant at the beginning (*r*_s_ = 0.818; *p* < 0.05), but not at the end of the experiment (*r*_s_ = 0.216; *p* > 0.05). In the mid temperature regime (b), the correlation between the duration of the behavior and the air temperature was significant at the beginning (*r*_s_ = 0.716; *p* < 0.05) and at the end of the experiment (*r*_s_ = 0.463; *p* < 0.05). In the low temperature regime (c), the number of beetles that exhibited the calling behavior was too small (*n* = 2) to perform statistical tests.

The above results indicate that in both the high (a) and moderate (b) temperature regimes, the calling behavior was performed with a significant tendency toward longer durations with higher air temperature. The duration tended to be longer if the beetle’s body temperature was higher. However, this tendency was only detected in the moderate temperature regime (b). Thus, male *R. batesi* appeared to require a certain temperature range for performing the calling behavior: the air temperature needs to be within the range of 26–33 °C, and the body surface temperature between 26–28 °C.

## 4. Discussion

In the present study, we report on the calling behavior of the male *Rosalia batesi*, and suggest an association between the peculiar abdominal tip morphology and the calling behavior. To the best of our knowledge, this is the first report of a putative pheromone secretion spot in the abdomen in the subfamily Cerambycinae. In this subfamily, the pheromone secretion spots are usually located in the thorax, as in many *Clytini* species [16,20,36] and in *Hylotrupes bajulus* (Callidiini) [37,38]. In line with this observation, males of the *R. batesi* species exhibit a very peculiar morphology of the abdominal tip when compared with other Japanese cerambycid species: the eighth abdominal tergite is less chitinized and is of a peculiar bifurcate shape, with numerous sensory apertures on the surface of the tergite; additionally, the chitinous eighth abdominal sternite is bifurcate in shape [33,34]. We observed the movement of this peculiar abdominal tip and speculated that this structure plays an important role in the calling behavior. Additionally, in the olfactometer experiments, in which odor-source animals were not visible, we perceived a peculiar odor when handling the male beetles, suggesting the emission of pheromones.

In an American congeneric species, *R. funebris*, male prothoracic glands and gland pores—which are rather common in cerambycine males [20,36]—are lacking, and the male pheromone source of this species has not yet been identified [20,21]. Considering these morphological and behavioral facts, namely the peculiar movement and structure of the eighth abdominal segment and the push-up stance, we assume that the secretion source of the volatile sex pheromone emitted by male *R. batesi* is on the eighth abdominal segment, with the pores on the eighth abdominal tergite serving as outlet points. We speculate that *Rosalia funebris* and *R. alpina* males share this morphology and ethology with *R. batesi*. Comparisons of the structure of the male abdominal tips of several *Rosalia* species are currently in progress to generalize these characteristics across the genus *Rosalia*, or at least the subgenus *Rosalia*.

As stated above, in the species studied thus far, the identified secretory sources of sex pheromones in the subfamily Cerambycinae (tribes Clytini and Callidiini) are the gland pores on the pronotum. Apart from this subfamily, *Prionus californicus* (Prioninae: Prionini) females often display lifting of the abdomen and expose a membranous cylindrical sac from the ovipositor, which is assumed to be pheromonal calling behavior [35,39]. *Tetropium fuscum* (Spondylidinae: Asemini) males also perform a calling behavior by raising their abdomen by 10°, suggesting that the abdominal tip is the pheromonal source [40]. This abdominal tip-protruding behavior in the pheromone-producing sex of the genera *Prionus* and *Tetropium* may be shared by *Rosalia*. However, the opening and closing motion of the abdominal tip is unique not only in the subfamily Cerambycinae but also in the family Cerambycidae.

In the T-shaped olfactometer experiments, the test female individuals, after arriving at the odor source (male), remained there motionless. In contrast, if the test females arrived at the control side or at another female as the odor source, they continued moving. This strongly suggests that the male’s presence rendered the female quiet even without physical contact with the male. This also supports the presence of a volatile sex pheromone in *R. batesi* that attracts only females.

In contrast, the male volatile pheromone of the American congeneric species, *R. funebris*, has been reported to attract both males and females, and has thus been designated “aggregation pheromone” [21]. Nevertheless, this pheromone lured mostly females (male–female = 1:11), suggesting that the substance is a sex pheromone rather than an aggregation pheromone. Cardé [23] propose the term “aggregation­–sex pheromone” for pheromones with dual function. Pheromones of another congeneric species from Europe, *R. alpina*, have also been classified as aggregation rather than sex pheromones on the basis of a field attraction study that resulted in a male–female ratio of 45:38 [24]. These two *Rosalia* species possess rather different pheromones ((*Z*)-3-decenyl (*E*)-2-hexenoate in *R. funebris* and 3,5-dimethyl-6-(1-methybutyl)-pyran-2-one in *R. alpina*); thus, their functions may differ. In the case of the genus *Xylotrechus* (Clytini), some species use the same chemical substance as an aggregation pheromone that another species uses as a sex pheromone, despite the fact that they share the prothoracic pheromone glands and chemical structure of the component(s) [32,41]. Another possible explanation for the different results on the function of pheromones in the different *Rosalia* species is the difference between laboratory and field experimental conditions. These conditions might have differed in terms of the age of the beetles and the pheromone concentration. Further research is needed to address this point. We are currently in the process of identifying the chemical structure of the male sex pheromone of *R. batesi*.

The results of the field observation of adult *R. batesi* are consistent with data published by Iwata et al. [10], which, to our knowledge, is the only previous study of the biology of *R. batesi*. Consistent with our observations, activity data from this previous study showed that *R. batesi* is typically diurnal, but this study did not differentiate behavior by sex. Therefore, the present study is the first to report the diurnal activity of both sexes. In the field observations, we found that adult male *R. batesi* were mostly active in the morning. Additionally, male beetles were often found in easily detectable spots, such as on the sunny, top surface of a log, suggesting that the males were sun-bathing. We speculate that males were heating up their bodies in the sun to be ready to emit sex pheromones to attract females during the appropriately warm hours of the day, when female beetles were also active. Our data on the temperature conditions necessary for males to perform the calling behavior (air temperature and body surface temperature between 26–33 °C and 26–28 °C, respectively) are consistent with this assumption. Thus, we suggest that temperature, in addition to the time of day, is an important factor for pheromone emission in *R. batesi*, in which male pheromone emission is assumed to take place around the noon. The other species, *Xylotrechus pyrrhoderus* (Cerambycinae: Clytini) have similarly been reported to show a peak in pheromone emission at 14:00 in laboratory experiments [42].

Among the male appeasement behaviors, we observed in *R. batesi*, the male’s biting of the female’s antenna (antenna-biting), followed by their pressing it down or fixing it to prevent spontaneous movement of the female—namely pressing down of the female antennae—appears to be unique among all known behaviors of Cerambycidae. In Lepturinae, the male bites the female’s antenna and presses it up, rather than down [27]. This behavior may be related to the difference in body size between males and females: Male *R. batesi* are, on average, larger than females. In contrast, in most other cerambycid species, the female is usually larger than the male.

## Figures and Tables

**Table 1:** Results of laboratory mating in *Rosalia batesi*. Before being subjected to the experiments, adult beetles were divided into groups of males and females, and into groups of larger and smaller than the average individuals.

Combination of the Male and the Female ^a)^	Individual No.		Body Length (mm)		Body Length Difference (Male—Female) (mm)	Time required until the First Mounting Took Place (sec)	Copulation Duration (sec) ^b)^					6th Copulation and so Forth
	Male	Female	Male	Female			1st	2nd	3rd	4th	5th	
Larger male vs. smaller female	M1	F1	29.5	22.1	7.4	1129	155	2	189	208	174	
	M2	F2	29.3	22.8	6.4	3600	96	195	–			
	M3	F3	33.0	23.3	9.7	15	–					
	M4	F4	30.2	23.9	6.3	3019	180	–				
	M4	F5	30.2	22.0	8.1	274	156	162	152	–		
Smaller male vs. smaller female	M5	F6	19.0	23.3	−4.2	1287	249	155	–			
	M6	F7	18.0	20.9	−3.0	578	214	91	285	33	97	yes
	M7	F8	21.8	22.8	−1.1	2112	110	242	174	290	220	yes
	M8	F9	20.9	22.0	−1.2	733	96	23	172	114	–	
	M9	F10	21.1	22.0	−0.9	134	221	74	46	98	148	yes
Smaller male vs. larger female	M7	F11	21.8	24.8	−3.0	671	212	15	91	140	55	yes
	M10	F12	21.3	27.1	−5.8	425	47	119	111	107	112	yes
	M11	F13	21.1	26.3	−5.2	1002	91	183	42	114	25	yes
	M8	F13	20.9	26.3	−5.5	45	211	37	147	125	208	
	M10	F13	21.3	26.3	−5.0	1449	121	136	132	–		
Larger male vs. larger female	M4	F13	30.2	26.3	3.8	1015	57	26	117	213	147	
	M12	F14	29.6	26.8	2.8	3242	166	26	170	–		
	M13	F12	26.6	27.1	−0.5	840	174	–				
	M14	F15	30.2	27.5	2.7	2867	122	100	18	105	–	
	M11	F13	28.0	26.3	1.6	1460	277	112	160	94	91	yes

^a)^ Larger and smaller males are those with more than, or less than, the average body length (24.5 mm), respectively. Larger and smaller females are those with more than, or less than, the average body length (24.7 mm), respectively. ^b)^ “–” means no more copulation took place thereafter.

**Table 2:** Appeasement behavior of males toward females in *Rosalia batesi*: the frequency of each behavior and its target.

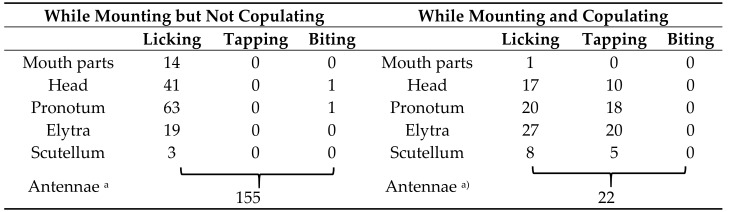

^a^ Since the attacks against antennae were vigorous and swift, attack items were not discriminated.

**Table 3:** Responses of male and female *Rosalia batesi* adults (test animals) to males or females (odor-source animals) as observed in the T-shaped olfactometer.

Odor-Source Animal	Test Animal	Responses (%)			Total (*N*)	χ^2^ Statistic
		(a) Odor-Source Animal Side	(b) Control Side (Blank)	(c) Inactive		χ^2^ (d.f. = 1) between (a) and (b)
male	male	38	52	10	50	1.0889 < χ^2^_0.05_ = 3.841
female	male	26	40	34	50	1.485 < χ^2^_0.05_ = 3.841
female	female	46	32	22	50	0.3141 < χ^2^_0.05_ = 3.841
male	female	84	8	8	50	31.3913 > χ^2^_0.001_ = 10.828
